# Supplementary material for: Simultaneous ventilation in the Covid-19 pandemic. A bench study
Source: PLoS One. 2021 Jan 19;16(1):e0245578. doi: 10.1371/journal.pone.0245578 (PMC7815120; doi:10.1371/journal.pone.0245578)
Supplement: Attachment — Submitted filename: Response to Reviewers.docx [file pone.0245578.s001.docx]

PONE-D-20-34127R1

Simultaneous ventilation in the Covid-19 pandemic. A bench study

PLOS ONE

Dear Dr. Guérin,

Thank you for submitting your manuscript to PLOS ONE. After careful consideration, we feel that it has merit but does not fully meet PLOS ONE’s publication criteria as it currently stands. Therefore, we invite you to submit a revised version of the manuscript that addresses the points raised during the review process.

One minor error to be corrected. Let us go one more round.

***R. Thank you very much for your appraisal of our revised version. The error disclosed has been corrected in the R2.***

Comments to the Author

Reviewer #1: The Authors have improved the manuscript ‘Simultaneous ventilation in the Covid-19 pandemic. A bench study’ and addressed all issues to my satisfaction.

***R. Thank you very much. Your words are highly appreciated.***

Reviewer #2: The authors have already addressed all of my comments and suggestions. I satisfy with the revised version. There is only one minor comment as follows:

- Page 7, 1st paragraph: please correct the unit of inspiratory flow setting from 60 "L/s" to be "L/min".

***R. Thank you very much. Your words are highly appreciated. Thank you for picking up this mistake. We have corrected 60 L/s to 60 L/min in the R2.***
